# Reliability of the Brazilian Portuguese version of the Gross Motor Function Measure in children with cerebral palsy

**DOI:** 10.1590/bjpt-rbf.2014.0131

**Published:** 2016-01-19

**Authors:** Kênnea M. Almeida, Karolina A. Albuquerque, Marina L. Ferreira, Stéphany K. B. Aguiar, Marisa C. Mancini

**Affiliations:** 1 Departamento de Educação Integrada em Saúde (DEIS), Universidade Federal do Espírito Santo (UFES), Vitória, ES, Brazil; 2 Curso de Fisioterapia, Universidade Federal de Minas Gerais (UFMG), Belo Horizonte, MG, Brazil; 3 Departamento de Terapia Ocupacional, Universidade Federal de Minas Gerais (UFMG), Belo Horizonte, MG, Brazil

**Keywords:** cerebral palsy, reproducibility of results, performance evaluation, rehabilitation, physical therapy

## Abstract

**OBJECTIVE:**

To test the intra- and interrater reliability of the Brazilian Portuguese version of the 66-item Gross Motor Function Measure (GMFM-66).

**METHOD:**

The sample included 48 children with cerebral palsy (CP), ranging from 2-17 years old, classified at levels I to IV of the Gross Motor Function Classification System (GMFCS) and four child rehabilitation examiners. A main examiner evaluated all children using the GMFM-66 and video-recorded the assessments. The other examiners watched the video recordings and scored them independently for the assessment of interrater reliability. For the intrarater reliability evaluation, the main examiner watched the video recordings one month after the evaluation and re-scored each child. We calculated reliability by using intraclass correlation coefficients (ICC) with their respective 95% confidence intervals.

**RESULTS:**

Excellent test reliability was documented. The intrarater reliability of the total sample was ICC=0.99 (95% CI 0.98-0.99), and the interrater reliability was ICC=0.97 (95% CI 0.95-0.98). The reliability across GMFCS levels ranged from ICC=0.92 (95% CI 0.72-0.98) to ICC=0.99 (95% CI 0.99-0.99); the lowest value was the interrater reliability for the GMFCS IV group. Reliability in the five GMFM dimensions varied from ICC=0.95 (95% CI 0.93-0.97) to ICC=0.99 (95% CI 0.99-0.99).

**CONCLUSION:**

The Brazilian Portuguese version of the GMFM-66 showed excellent intra- and interrater reliability when used in Brazilian children with CP levels GMFCS I to IV.

## Introduction

The Gross Motor Function Measure (GMFM) is a standardized, valid, reliable, and responsive tool designed to evaluate gross motor function in children with cerebral palsy (CP)^1^^,^ 2. This measure has been widely used in research and clinical practice in the field of child rehabilitation in different countries and has served as a reference for the development of other tests and classification systems^3^^-^ 8.

The GMFM aims to measure gross motor function, to help define therapeutic goals, to record changes over time, to inform caregivers of the progress made in the rehabilitation process, and to enable the development of scientific research studies in the field^1^^,^ 2. Following training, the measure should be used preferably by pediatric physical therapists and occupational therapists^1^.

The first version of the GMFM consisted of 88 items divided into five dimensions, namely, dimension A - lying and rolling (17 items); dimension B - sitting (20 items); dimension C - crawling and kneeling (14 items); dimension D - standing (13 items); and dimension E - walking, running, and jumping (24 items)^1^. The score of each item is based on a four-point scale, where a score of zero (i.e. "*does not initiate* ") means that the child is unable to start any activity and a score of three (i.e. *"complete"* ) informs that the child completes 100% of the activity tested by the item; intermediate scores (i.e. scores of 1 and 2) describe partial performances of the item^1^. The GMFM includes two types of items, dynamic and static. In the dynamic items, the examiner observes the child's movements (e.g. item 78: *standing* , where the child must kick a ball with the right foot). In the static items, the focus is on the child maintaining the initial position for a specific period of time (e.g. item 39: *the child must maintain the weight on hands and knees for 10 seconds* ). The description of the expected behavior for each score is detailed in the GMFM's manual^1^. The scores of all items are added after completing the test and are converted into a percentage performance^1^. The GMFM-88 version is also used to evaluate children with syndromes and other disorders affecting motor development^9^^-^ 11.

A second version with 66 items (GMFM-66) was developed and tested using the GMFM-88 data from 537 children, making it possible to undertake Rasch analyses and extract a 66-item version^1^^,^ 12. The original 88 items were downsized mainly by excluding items from dimensions A (reduced from 17 to four items) and B (from 20 to 15 items)^1^. Rasch analysis redistributed the items over a continuous range of relative difficulty, which enables the professionals using the test to identify items within each child's specific functional range^12^^,^ 13. The testing procedures and scoring are conducted as in the longer version. After completing the test, the scores of all items are plotted using the Gross Motor Ability Estimator (GMAE) software, which converts the result into a scale ranging from zero to 100 and provides a map of tested items ranked by the degree of relative difficulty^1^.

The GMFM-66 requires a shorter application time (approximately 45 minutes), enables the examiner to calculate the total score, even if some items are not tested, and is the recommended version for research purposes. This new version should only be used with children at levels I to IV of the Gross Motor Function Classification System (GMFCS). Children at level V should only be evaluated using the GMFM-88 because it includes more items of lower complexity^1^^,^ 2.

The GMFM validation sample was collected in Canada and consisted of 111 children with CP, 25 with brain injury, and 34 children under five years of age with normal motor development^1^^,^ 2. Content validity was tested by a group of experts in pediatric developmental assessment, who selected items corresponding to the gross motor skills of a five-year-old child without motor impairment to integrate the test content^1^. Inter- and intrarater reliability values were tested in 12 children with CP and evaluated by six experienced therapists. The results, assessed using the intraclass correlation coefficient (ICC), showed excellent test consistency (ICC=0.99 for both)^1^. Intra- and interrater reliability values were also assessed in each test dimension, with results ranging from 0.87 to 0.99; the lowest values were detected in the interrater reliability of dimension A^1^.

GMFM-66 shows psychometric characteristics similar to those of GMFM-88^1^^,^ 12 ^-^ 14. The reliability of the new version was tested in some studies and consistently presented excellent estimates. A study conducted by the authors of the instrument^2^ on 19 children with CP, who were all evaluated by the same rater, reported an intrarater reliability of ICC=0.99. Wei et al.^15^ assessed the GMFM-66 intra- and interrater reliability values of two raters in a sample including 20 children with CP aged between zero and three years and also observed excellent reliability, with ICC=0.97 and ICC=0.98, respectively.

The first requirement for a good standardized test is reliability, that is, the extent to which a measure is consistent and error-free, without which reliable data cannot be collected and inferences cannot be made from the data^16^^,^ 17. Reliability is not a fixed feature but is rather the product of interactions between instruments, raters and subjects in the evaluation context^18^. The main types of reliability reported in the literature are interrater reliability, which is the estimate of how consistent the test is when applied by different raters using the same scale to assess the same subjects or objects, and intrarater reliability, which informs on the consistency of scores when individuals are assessed on two or more occasions by the same rater, using the same scale^16^^-^ 18.

The authors are responsible for providing the initial test validity and reliability data. However, those characteristics are never assessed definitively, as continuous evaluations of psychometric properties are required^17^^,^ 19. Evidence of the validity and reliability of an instrument does not guarantee that it will be used validly and reliably, particularly in a population culturally different from the population in which it was developed^17^. Thus, an instrument's psychometric properties must be tested each time a scale is used in a new context or with a different group of people.

The GMFM manual and score sheet were translated into Brazilian Portuguese^20^. To the best of our knowledge, the psychometric properties of the translated instrument have not been tested yet in the Brazilian population, though the original untranslated version is often used in research studies conducted in Brazil^21^. The assessment of GMFM reliability may show local parameters for the use of the instrument in clinical practice and research studies conducted in the country. This study aimed to assess the intra- and interrater reliability values of the Brazilian Portuguese version of the GMFM-66 in CP children with GMFCS levels ranging from I to IV.

## Method

### Sample

A total of 48 children diagnosed with CP were selected from clinics and rehabilitation centers between January 2013 and July 2014. The study was approved by the Human Research Ethics Committee of Universidade Federal de Minas Gerais (UFMG), Belo Horizonte, MG, Brazil (protocol no. 476.437). The parents or guardians were invited to participate and were informed about the study procedures. Those who voluntarily agreed to their child's participation in the study signed an informed consent form.

Participants were children with CP diagnosis (GMFCS levels I to IV) confirmed by medical report and with the ability to understand and follow simple verbal commands. The sample size was calculated using the equation derived from Pearson's product-moment correlation, as reported by Streiner^22^, considering α=0.05, a 95% confidence interval (CI) and an expected ICC>0.90^20^, which resulted in 48 children, including 12 in each GMFCS group^17^^,^ 22.

Four raters also participated in this study, including three physical therapists and one occupational therapist, who work in the field of pediatric rehabilitation. Only one of these raters was experienced in using the GMFM. Three raters had neither experience in using the instrument nor prior access to the English manual, not even during the study.

### Procedures

The raters were trained in the use of the GMFM by two therapists with experience using the instrument. The training consisted of reading the manual translated into Portuguese and discussing test application videos with experienced therapists for 12 hours. After training, one of the raters was selected to be the primary rater and to administer the GMFM to all of the children. The other raters watched the footage to assess interrater reliability. The primary rater had previously applied the GMFM-66 to children with normal motor development as a practical exercise to become more familiar with the score sheet and test application guidelines, as suggested by the manual^20^.

The evaluations were performed in rehabilitation centers, locations well-known to the children, in spacious rooms at least five meters long, using benches, stairs, a stopwatch, sticks and drawing lines, and circles on the ground to apply specific items^20^. The primary rater evaluated each child only once and filled out the test score sheet at the time of on-site assessment, consulting the manual whenever necessary. The children were evaluated while wearing comfortable clothes and no shoes and without using any assistive devices or orthotics. The items administered to each child were those whose completion was considered feasible by the rater, who allowed three attempts toward identifying the best performance^20^.

All evaluations were recorded using a digital video camera according to a predefined standardized method. For most items applied, the camera was placed on a tripod and was positioned between the frontal and sagittal planes, according to the type of movement to be recorded, so that the child's entire body or body part to be examined was visible to the camera. Items requiring a wider camera angle, namely, items including walking, were recorded with the camera on the tripod, and its digital zoom was controlled by a research assistant. Descriptive data were collected on the same day for each child, including gender, age, social class, and GMFCS level. The entire procedure lasted 50 to 60 minutes^20^.

To assess intrarater reliability, the primary rater watched the footage and filled out the score sheet again four weeks after the on-site assessment. This period was necessary to avoid recall bias of the on-site assessment scoring^16^. The scores obtained in the assessments were transferred into the GMAE, wherein the final score of the GMFM-66 assessment was generated for each child. To assess interrater reliability, the other three raters received the randomized videos recorded during the assessment of each child, including information on the administered items. The blinded raters, with no access to the sample data (GMFCS and age, among others), watched the footage of the 48 children selected, filled out the GMFM score sheets independently, and entered the data in the GMAE software.

### Statistical analysis

All results assessed in the data collection were entered into the Statistical Package for the Social Sciences (SPSS) version 20.0. The values for intra- and interrater reliability regarding the total sample and each GMFCS group were calculated based on the final score generated using the GMAE. The reliability of each GMFM dimension was also calculated based on the net score. A measure of consistency (ICC type 3.1) with two-way mixed analysis^23^ was used to assess intrarater reliability. A measure of absolute agreement with two-way random analysis between the scores obtained in the assessments conducted by the primary rater and the other raters (ICC type 2.1)^23^ was applied to assess interrater reliability.

The ICC is an estimate ranging from zero (unreliable measure) to one (perfect reliability), assessed by the ratio between inter-group variance (including random error) and total variance. The ICC is a reliability parameter appropriate for measuring the agreement or consistency between two or more interval measures^24^. The results of correlation coefficients are interpreted as follows: weak or no correlation from zero to 0.25; fair correlation from 0.25 to 0.50; moderate correlation from 0.50 to 0.75; and very good to excellent correlation for values higher than 0.75^25^. However, the interpretation of reliability based on the ICC values varies according to the specificity of each study. A more stringent criterion is recommended, considering an ICC minimum value of 0.90, when a test is used for clinical decision-making for individuals with specific health conditions^18^^,^ 24.

## Results

A total of 48 children with CP, aged two to 17 years old, primarily from families of lower socio-economic classes and predominantly with spastic-type of CP, participated in this study. Table 1 outlines the main descriptive characteristics of the sample according to the GMFCS level. The mean scores obtained in the first assessment performed by the primary rater in each GMFCS group indicated that the higher the group severity is, the lower the score obtained in the GMFM-66 will be.

**Table 1:** - Descriptive characteristics of the total sample and per GMFCS group.

Description	GMFCS I	GMFCS II	GMFCS III	GMFCS IV	Total sample
Number of children	12	12	12	12	48
Age (mean)	7.75	8.58	8.42	10.41	8.8
Gender (n)					
Female	8	2	6	9	25
Male	4	10	6	3	23
Clinical Type (n)					
Spastic hemiplegia	8	2	0	0	10
Spastic diplegia	4	7	9	2	22
Spastic quadriplegia	0	1	1	9	11
Dyskinetic	0	1	1	1	3
Ataxic	0	0	1	0	1
Mixed	0	1	0	0	1
GMFM-66 mean score	78.53	63.75	53.47	41.21	-

GMFCS=Gross Motor Function Classification System; GMFM=Gross Motor Function Measure; n=frequency.

To calculate reliability, 48 on-site assessments and 192 video reviews were performed. Table 2 shows the results of intra- and interrater reliability rates in the total sample, in the GMFCS groups, and in the five dimensions of the GMFM-66. The GMFM-66 scores showed excellent intra- and interrater reliability rates (ICC 0.99 and 0.97, respectively) when the total sample was analyzed. The reliability rates of results assessed in each GMFM-66 dimension were also considered excellent, all with ICC>0.95. 

**Table 2:** - Intra- and interrater reliability rates.

GMFM-66 structure	N	Intrarater reliability ICC_3,1_ (95%CI)	Interrater reliability ICC_2,1_ (95%CI)
GMFM-66 (GMAE) total score	48	0.99 (0.98-0.99)	0.97 (0.95-0.98)
Result per subscale			
Dimension A	48	0.98 (0.97-0.99)	0.95 (0.93-0.97)
Dimension B	48	0.99 (0.98-0.99)	0.96 (0.94-0.98)
Dimension C	48	0.99 (0.99-0.99)	0.99 (0.98-0.99)
Dimension D	48	0.99 (0.99-0.99)	0.99 (0.99-0.99)
Dimension E	48	0.99 (0.99-0.99)	0.99 (0.99-0.99)
GMFM-66 total score (GMAE) per GMFCS			
GMFCS I	12	0.98 (0.95-0.99)	0.93 (0.84-0.98)
GMFCS II	12	0.99 (0.97-0.99)	0.98 (0.96-0.99)
GMFCS III	12	0.99 (0.99-0.99)	0.99 (0.99-0.99)
GMFCS IV	12	0.92 (0.72-0.98)	0.94 (0.84-0.98)

N=number of subjects; GMFM=Gross Motor Function Measure; GMAE=Gross Motor Ability Estimator; GMFCS=Gross Motor Function Classification System; ICC=Intraclass Correlation Coefficients; CI=Confidence Interval.

The reliability remained excellent in the four GMFCS groups, with the lowest value detected for the intrarater reliability of GMFCS IV (ICC=0.92), which also showed the widest 95% CI (0.72 to 0.98).

## Discussion

The present study demonstrated that the Portuguese-translated version of the GMFM-66 has excellent intra- and interrater reliability when applied to Brazilian children with CP, GMFCS levels I to IV. This result indicated that the Brazilian version corroborates the reliability rates of the original version of the test, as assessed by Russell et al.^1^^,^ 2 ^,^ 20.

The GMFM has been translated into different languages in various countries, and the psychometric properties of the translated versions have been consistently assessed^4^^,^ 15 ^,^ 26. The interrater reliability of the Korean version was assessed in 39 children with CP by two experienced raters following training to use the test^4^. The Korean version also showed excellent interrater reliability in the GMFM dimensions, with the ICC values ranging from 0.98 in dimension A to 0.99 in dimension E^4^. Subsequently, the same authors assessed the reliability in 84 children with CP through video reviews conducted by 10 therapists who were trained for 30 hours. The authors identified excellent interrater reliability in all GMFM dimensions (ICC values ranging from 0.97 to 0.99) and excellent intrarater reliability (ICC values ranging from 0.99 to 1.00)^27^.

Mahasup et al.^26^ assessed the intra- and interrater reliability rates of the GMFM in 10 Thai children with CP. Three raters participated in the study, including one experienced in using the GMFM and two raters who read the manual and received training. On-site assessments were performed by the experienced rater and were recorded to enable the other raters to review them and score the children independently using the footage. The study showed excellent intrarater (ICC=0.99) and interrater (ICC=0.93) reliability rates regarding the total sample score^26^. The present study is similar to the others cited above^2^^,^ 4 ^,^ 26 regarding the sample characteristics of both children and raters and regarding the methods and results. Furthermore, consistency between the literature studies and the present study was also observed in terms of the previous training of raters and the use of the manual during the application of the test^2^^,^ 4 ^,^ 26 ^,^ 27. Such methodological consistency enables the comparison of results.

The evaluation of child development using standardized instruments is complementary to purely observational clinical evaluations because such a method is structured toward minimizing subjective interpretations and ensuring the consistency of the results by assessing the psychometric properties of the tests^19^. The reliability of a test refers to its capacity for providing consistent results. Several factors may affect the agreement of scores, including the evaluation setting, the psychological status and age of the examinee, familiarity between examinee and rater and, especially, the knowledge, experience, and skills of the raters^16^^,^ 17 ^,^ 28. It is important to conduct studies that evaluate different types of reliability, to cover as much as possible the sources of error^16^^,^ 17 ^,^ 19. According to the methodology used in the present study each child was evaluated with the GMFM only once, this administration was video recorded, and evaluations were scored again from the videotaped assessments. The results showed that examiners trained to look at children with CP on the GMFM items can consistently score them (i.e. interpret the performances). However, our study did not test the test-retest reliability, in which the examiner's ability to apply the test and get a similar performance in the scores would be checked by applying the GMFM more than once to the same individuals under similar conditions. Future reliability studies should investigate the examiner's skill in using the test as a source of variability for the Portuguese version.

The training of raters in the administration and scoring of observational instruments is a major factor in the consistent administration of these instruments and a key strategy for helping to minimize errors^1^^,^ 19 ^,^ 20. Russell et al.^28^ showed that raters improve the agreement of their scores after GMFM training workshops and that the training process has a greater effect on the ability to learn and administer the test than years of pediatric clinical experience. The sample of raters in the present study included four therapists; three of them, including the primary rater, had no prior experience in using the test and had worked in pediatric rehabilitation for less than one year. However, all raters were properly trained, according to instructions in the manual, by two experienced therapists, and the results indicate that the GMFM-66 can be used by new Brazilian therapists, provided they have received previous training. The study by Ko and Kim^27^ assessed the interrater reliability of the GMFM Korean version between one experienced rater and one inexperienced rater and identified excellent reliability in all five GMFCS levels, which corroborates the results from the present study.

The high ICC values measured in this study may also be explained by the use of video footage to assess reliability. The videos enable the raters to watch the items as many times as necessary and to pause the videos to review the scoring guidelines in the manuals, resulting in greater reliability^4^. Video review is a rather objective form of evaluation, wherein the children's performance, rather than their ability to perform a task, is observed^20^. This strategy may be used in both clinical practice and research studies aiming to increase the reliability of assessments using the GMFM.

This study aimed to assess the reliability of the GMFM-66, which is the version that best enables the longitudinal quantification of changes in gross motor function of children with CP^12^^-^ 14. The improvement of the test as a longitudinal assessment tool was enabled by the Rasch analysis, which reorganized the items in a continuum of difficulty, showing the hierarchical structure of the instrument and providing information about the prior and emergent motor functions of each child^12^. The Rasch analysis also allowed one of the requirements for using parametric statistical tests in research studies to be met by transforming the GMFM-66 total score into an interval scale^12^. However, the GMFM-66 may only be administered in children with CP, preferably with GMFCS levels I to IV, as in this study sample. The reliability of the GMFM Brazilian version should also be tested encompassing all test items by applying the GMFM-88 to children with different health conditions to whom this version of the test may be applied.

The original GMFM version has been used in Brazilian research studies mainly aimed at assessing the effects of interventions on the motor functions of children with CP^21^^,^ 29 ^,^ 30. The translation of the manual and evidence of reliability in the Brazilian population of children with CP will enable the use of this test to be expanded to research and clinical practice. The raters reported no difficulties in using the translated versions, and the training sufficed to apply the test. However, some translation errors were identified by experienced examiners with prior knowledge of the original version. For example, the description of score 3 of one item is defined in the test score sheet as "incomplete" instead of "fully complete", which may confuse the inexperienced rater^20^. Another issue is the lack of translation of the GMAE software into Portuguese, despite coming with the manual, which will require therapists seeking to use the GMFM-66 to have some knowledge of the English language. These and other minor adjustments may be included in the new Portuguese edition of the manual.

## Conclusion

The GMFM-66 version, translated into Portuguese, shows excellent intra- and interrater reliability values when used in children with CP between GMFCS levels I and IV and may be used in clinical practice and in research studies on Brazilian children.
